# Supplementary material for: Over-activation of a nonessential bacterial protease DegP as an antibiotic strategy
Source: Commun Biol. 2020 Oct 1;3:547. doi: 10.1038/s42003-020-01266-9 (PMC7529758; doi:10.1038/s42003-020-01266-9)
Supplement: Supplementary file 1 — Supplementary Information [file 42003_2020_1266_MOESM1_ESM.pdf]

Supplementary Information

Over-activation of a nonessential bacterial protease DegP as an antibiotic strategy

Hyunjin Cho, Yuri Choi, Kyungjin Min, Jung Bae Son, Hyojin Park, Hyung Ho Lee and Seokhee Kim*

Department of Chemistry, Seoul National University, Seoul 08826, Republic of Korea

Table of contents

Supplementary Figures………………………………………………………………………………..S3

Figure S1. MALDI spectra of the tripodal peptidyl compounds……………………………………………………S3

Figure S2. The C-terminal isoleucine in TMB_CYYKI is critical for the high-affinity activation effect………….S4

Figure S3. Tripodal compounds show different allosteric effects…………………………………………….S5-S6

Figure S4. Full images of SDS-PAGE gels shown in Fig. 2e……………………………………………………….S7

Figure S5. TMB_CYYKI functions as a permanent activator………………………………………………………S8

Figure S6. The tripodal compounds can kill the OM-permeable *E. coli*………………………………………….S9

Figure S7. The combination of TMB_CYYKI with chloramphenicol disrupts the killing effect…………….S10

Figure S8. Co-crystal structures of DegP^{S210A} and the tripodal compounds…………………………S11-S12

Figure S9. Growth inhibition test of DMB_CYYKI and MMB_CYYKI at 30°C or 42°C………………………..S13

Supplementary Tables………………………………………………………………………………….S14

Table S1. Predicted and observed masses for various compounds from MALDI…………………………….S14

Table S2. Bacterial strains used in this study……………………………………………………………………….S15

Table S3. Plasmids used in this study……………………………………………………………………………….S15

Supplementary References……………………………………………………………………….S16

Supplementary Figures

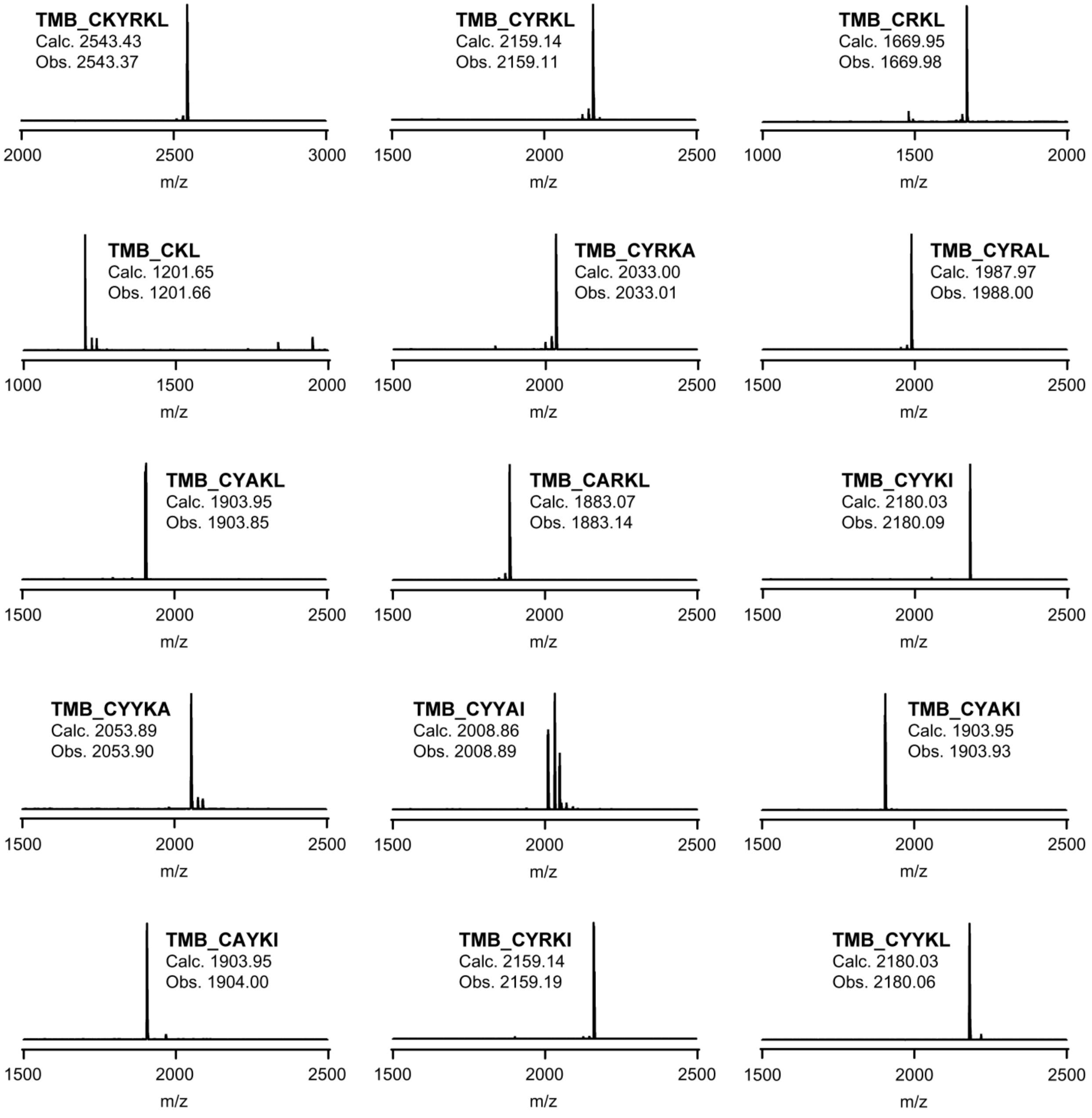

Supplementary Figure 1. MALDI spectra of the tripodal peptidyl compounds. See Supplementary Table 1 for comparison of calculated and observed molecular weights.

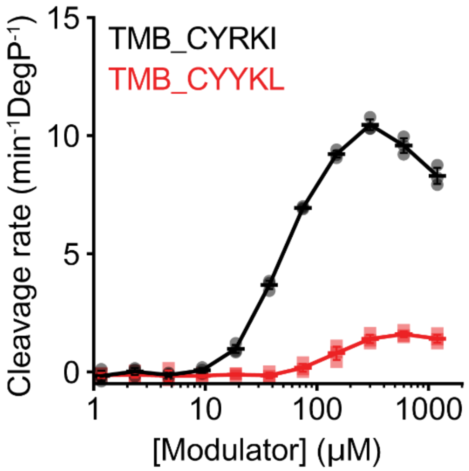

Supplementary Figure 2. The C-terminal isoleucine in TMB_CYYKI is critical for the high-affinity activation effect. Activation effects of compounds were monitored by measuring cleavage rates of the reporter peptide (100 μM) by DegP (1 μM) in the presence of increasing concentrations of TMB_CYYK**L** (green) or TMB_CYRK**I** (blue). Data are presented as dot plots with mean ±1 SD (n = 3 independent experiments).

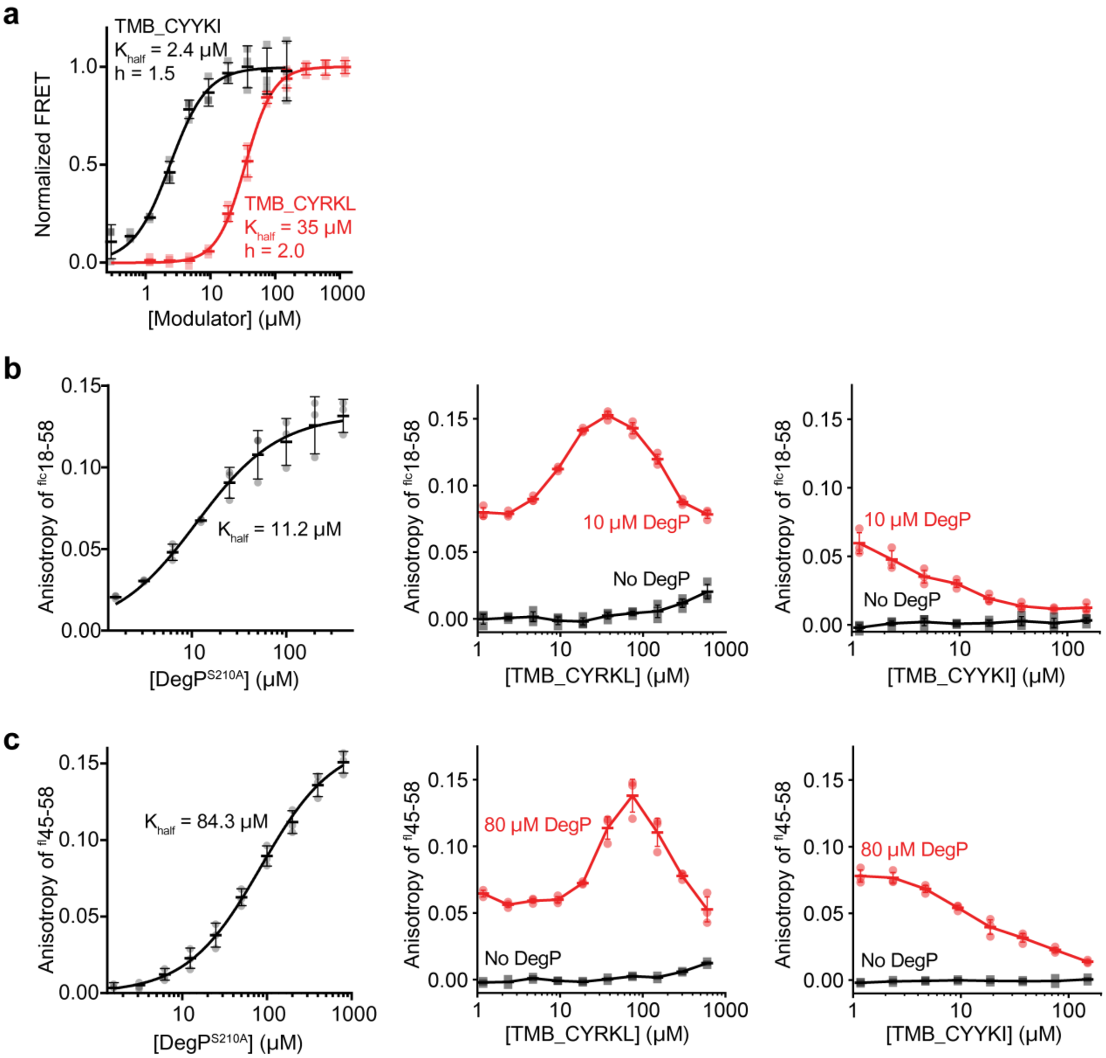

Supplementary Figure 3. Tripodal compounds show different allosteric effects. (a) DegP assembly was monitored by measuring the fluorescence resonance energy transfer (FRET) of the donor- and acceptor-labeled DegP variants (0.1 μM) with increasing concentrations of TMB_CYYKI or TMB_CYRKL. Data were fitted to the Hill equation ($y = [L]^h/(K_{half}^h + [L]^h)$). Data are presented as dot plots with mean ±1 SD (n = 3 independent experiments). **(b)** Anisotropy of the fluorescently labeled 18-58 (^{flC}18-58; 0.05 μM) was monitored with increasing concentrations of DegP^{S210A}. Data were fitted to a hyperbolic curve ($y = [L]/(K_{half} + [L])$), resulting in K_{half} = 11.2 μM (left). Therefore, about half of ^{flC}18-58 binds to DegP^{S210A} when [DegP^{S210A}] = 10 μM. The anisotropy of the fluorescently labeled 18-58 (^{flC}18-58; 0.05 μM) was monitored in the absence or presence of DegP^{S210A} (10 μM) with increasing concentrations of TMB_CYRKL (middle) or TMB_CYYKI (right). Data are presented as dot plots with mean ±1 SD (n = 3 independent experiments). (**c**) Anisotropy of the fluorescently labeled PDZ1 binder (^{fl}45-

58; 0.05 μM) was monitored with increasing concentrations of DegP^{S210A}, and analyzed as in Supplementary Fig. 3b, resulting in K_{half} = 84.3 μM (left). Anisotropy of the fluorescently labeled PDZ1 binder (^{fl}45-58; 0.05 μM) was monitored in the absence or presence of DegP^{S210A} (80 μM) with increasing concentrations of TMB_CYRKL (middle) or TMB_CYYKI (right). Data without DegP^{S210A} (negative control) are displayed only as dot plots (n = 2), but data with DegP^{S210A} are presented as dot plots with mean ±1 SD (n = 3 independent experiments)

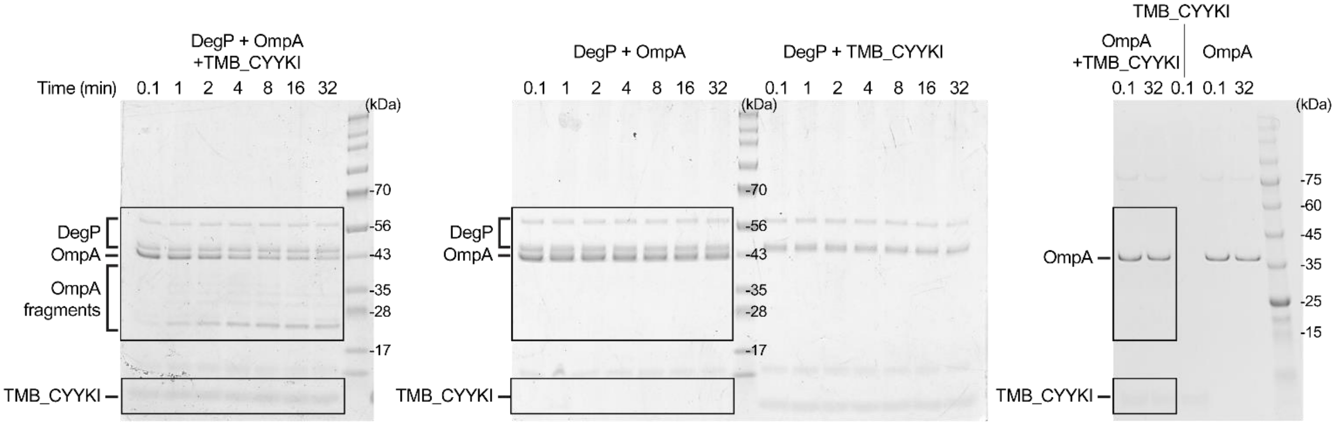

Supplementary Figure 4. Full images of SDS-PAGE gels shown in Fig. 2e.

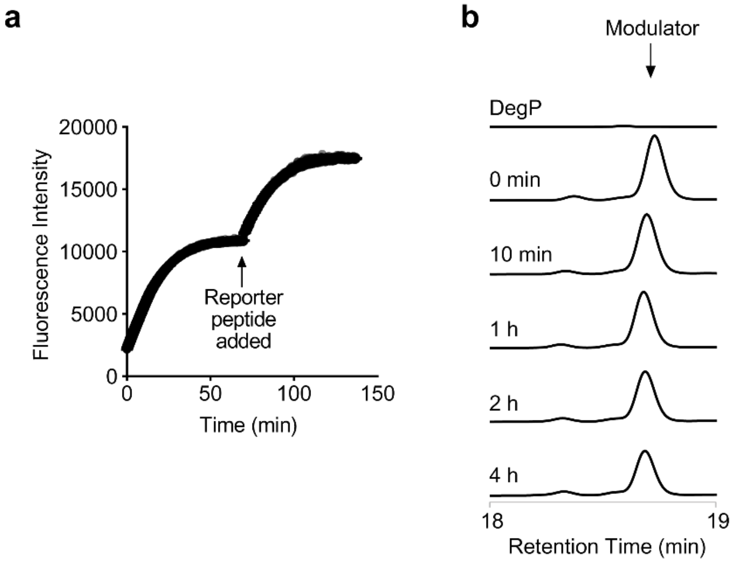

Supplementary Figure 5. TMB_CYYKI functions as a permanent activator. (a) Reporter (100 μM) cleavage by DegP (1 μM) in the presence of TMB_CYYKI (10 μM) was monitored for about 70 minutes, at which additional reporter (100 μM, final) was added and the cleavage was further monitored for additional 70 minutes. TMB_CYYKI-mediated activation of DegP is intact. Data are presented as dot plots with mean ±1 SD (n = 3 independent experiments). **(b)** TMB_CYYKI (10 μM) was incubated with DegP (10 μM) and samples taken at indicated times were analyzed by HPLC. TMB_CYYKI is not cleaved by DegP.

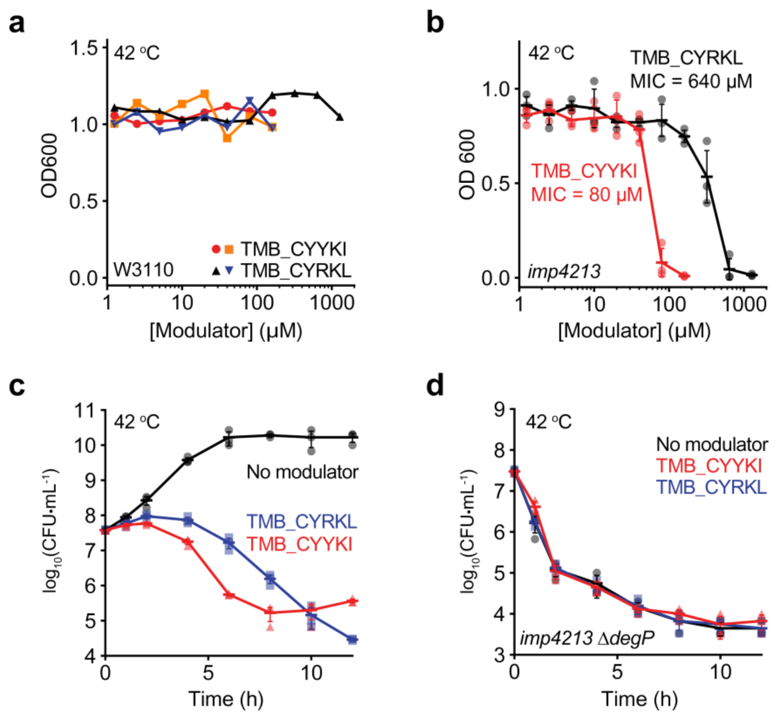

Supplementary Figure 6. The tripodal compounds can kill the OM-permeable *E. coli*. (a) Growth of the wild-type W3110 cells in the presence of TMB_CYYKI or TMB_CYRKL. Overnight cultures in LB broth were diluted 100-fold in the presence of different concentrations of TMB_CYYKI or TMB_CYRKL. Growth was monitored by OD_{600} after 12-hour incubation at 42°C. Cells with intact outer membrane (W3110) grow normally in the presence of the tripodal compounds. **(b)** Growth of the *imp4213* cells in the presence of TMB_CYYKI or TMB_CYRKL at 42°C. Data are presented as dot plots with mean ±1 SD (n = 3 independent experiments). **(c)** Time-kill curves of *E. coli imp4213* strain with TMB_CYYKI, TMB_CYRKL or no compound. Cells at log phase (OD_{600} = 0.01) were incubated at 42°C in the absence or presence of TMB_CYYKI or TMB_CYRKL (160 μM and 1280 μM, respectively). Cells at different times were taken out to determine colony forming units (CFUs). Data are presented as dot plots with mean ±1 SD (n = 3 independent experiments). **(d)** Time-kill curves of the *imp4213* Δ*degP* cells with TMB_CYYKI, TMB_CYRKL, or none of them at 42°C. The *imp4213* Δ*degP* cells are killed by heat shock. Data are presented as dot plots with mean ±1 SD (n = 3 independent experiments).

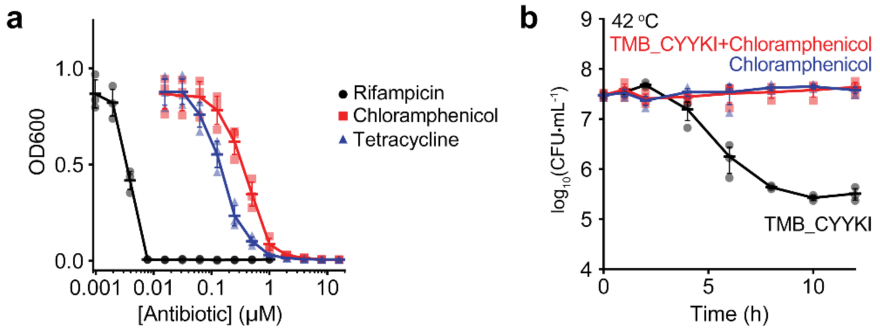

Supplementary Figure 7. The combination of TMB_CYYKI with chloramphenicol disrupts the killing effect. (a) MIC values of various chloramphenicol, tetracycline and rifampicin were determined by monitoring growth of the *imp4213* cells at 30°C in the presence of different concentrations of antibiotics. MIC values are: chloramphenicol, 2 μg/ml; tetracycline, 1 μg/ml; rifampicin, 0.008 μg/ml. Data are presented as dot plots with mean ±1 SD (n = 3 independent experiments). **(b)** Time-kill curve of the *imp4213* strain with TMB_CYYKI (160 μM), chloramphenicol (20 μg/ml), or both at 42°C. Data are presented as dot plots with mean ±1 SD (n = 3 independent experiments).

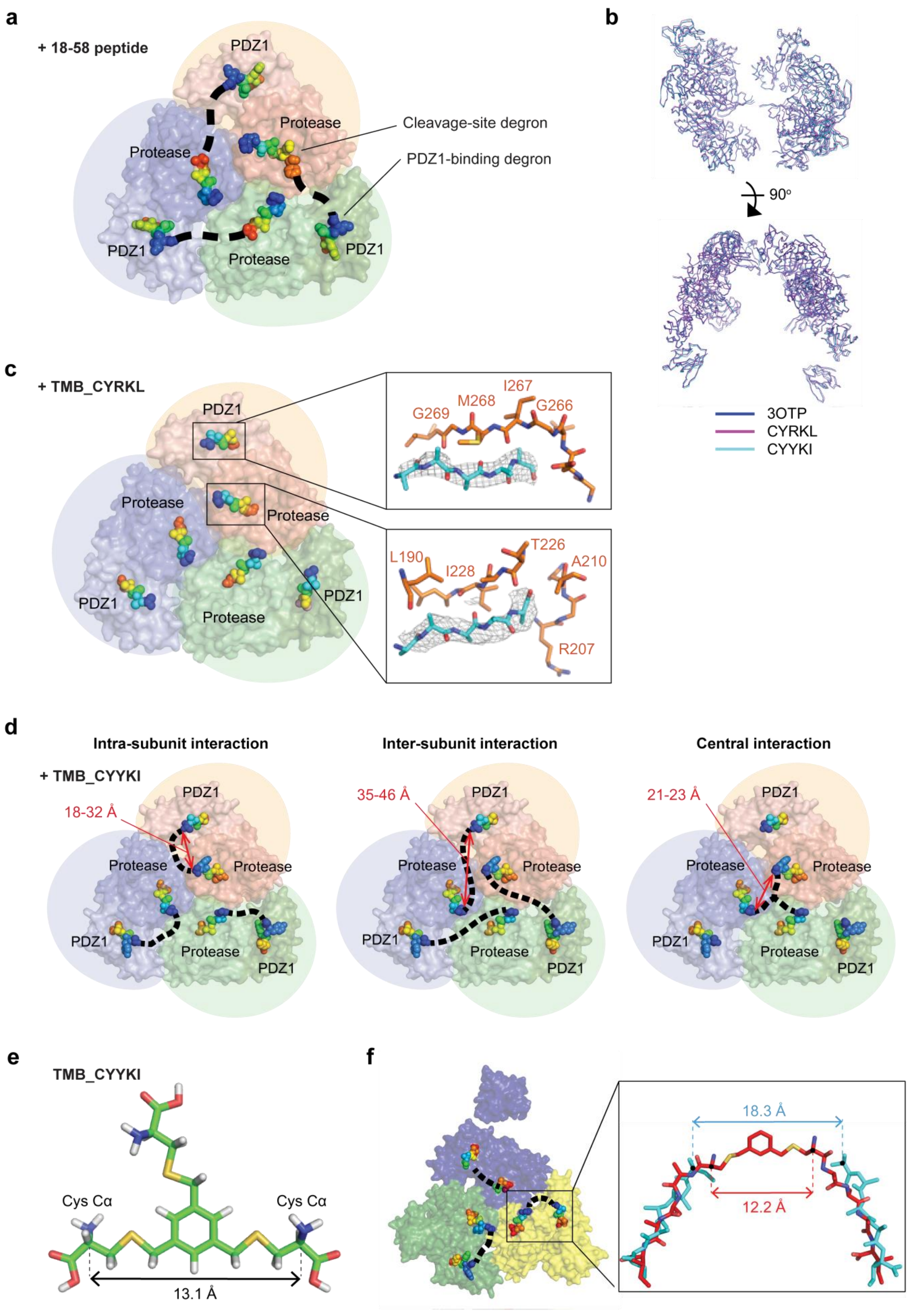
a
+ 18-58 peptide
PDZ1
Protease
Cleavage-site degron
PDZ1-binding degron
Protease
PDZ1
Protease
PDZ1
b
90°
3OTP
CYRKL
CYYKI
c
+ TMB_CYRKL
PDZ1
G269
M268
I267
G266
Protease
Protease
L190
I228
T226
A210
R207
PDZ1
PDZ1
Protease
d
Intra-subunit interaction
Inter-subunit interaction
Central interaction
+ TMB_CYYKI
18-32 Å
35-46 Å
21-23 Å
PDZ1
Protease
Protease
PDZ1
Protease
PDZ1
e
TMB_CYYKI
Cys Cα
Cys Cα
13.1 Å
f
18.3 Å
12.2 Å

Supplementary Figure 8. Co-crystal structures of DegP^{S210A} and the tripodal compounds. (a) A molecular model of a trimer of the dodecameric DegP with bound 18-58 (PDB code, 3OTP). Three DegP monomers (surface presentation) are colored differently and two degrons of 18-58 (spheres) are colored by the rainbow scheme (from the blue N-terminus to the red C-terminus). The most probable connection of two degrons in 18-58 is shown with a dashed line, in which the C-terminus of a cleavage-site degron is connected to the N-terminus of a PDZ1-binding degron that binds to the neighboring DegP subunit (inter-subunit connection). **(b)** Superposition of six crystallographically independent DegP subunits from the previously reported dodecameric structure (PDB code, 3OTP)[1] and our structures with TMB_CYRKL or TMB_CYYKI. **(c)** The molecular model of DegP^{S210A}•TMB_CYRKL drawn similarly to Fig. 5b. **(d)** Three possible binding modes in which at least two peptidyl arms in one compound occupy the substrate-binding sites; intra-subunit interactions, inter-subunit interactions, or central interactions. In the first mode with intra-subunit interactions, two arms occupy both the active-site region and the PDZ1 domain in one DegP monomer (left). The second mode with inter-subunit interactions is similar to that of 18-58, in which the two arms occupy the active site of one subunit and the PDZ1 domain of the neighboring DegP subunit (middle). The last mode has central interactions in which three arms occupy three active sites of the trimer (right). However, our crystal structures indicate that none of them are feasible, because the distances between two cysteine-Cα atoms in the CYYKI peptides of the crystal structure (18-32 Å, 35-46 Å, and 21-23 Å, respectively, for three modes) are much longer than that in the extended tripodal compound (13.1 Å; see Supplementary Fig. 8e). **(e)** The extended conformation of the tripodal compound shows that the maximal distance between two cysteine Cα atoms is about 13.1 Å. **(f)** A model of the compound with two peptidyl arms for intra-subunit interactions. The slight change of the N-terminal position of the two peptidyl arms, albeit with some energetic cost from the loss of β-sheet-type hydrogen-bonds, allows to connect them to the benzylic scaffold without changing the position of C-terminal residues (red sticks). Blue sticks present the crystal structure of DegP^{S210A}•TMB_CYYKI.

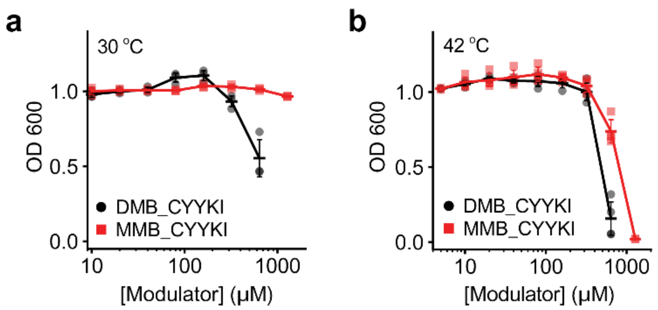

Supplementary Figure 9. Growth inhibition test of DMB_CYYKI and MMB_CYYKI at 30°C or 42°C. Growth of the *imp4213* cells at 30°C **(a)** or 42°C **(b)** in the presence of different concentrations of DMB_CYYKI or MMB_CYYKI. Data are presented as dot plots with mean ±1 SD (n = 3 independent experiments).

Supplementary Tables

Supplementary Table 1. Predicted and observed masses for various compounds from MALDI.

Compound	Calculated $[M + H]^+$	Observed $[M + H]^+$	Δ	ppm
TMB_CKYRKL	2543.43	2543.37	0.06	24.38
TMB_CYRKL	2159.14	2159.11	0.03	17.23
TMB_CRKL	1669.95	1669.98	0.03	17.25
TMB_CKL	1201.65	1201.66	0.01	11.00
TMB_CYRKA	2033.00	2033.01	0.01	2.71
TMB_CYRAL	1987.97	1988.00	0.03	15.69
TMB_CYAKL	1903.95	1903.85	0.10	56.30
TMB_CARKL	1883.07	1883.14	0.07	37.92
TMB_CYYKI	2180.03	2180.09	0.06	28.07
TMB_CYYKA	2053.89	2053.90	0.01	4.33
TMB_CYYAI	2008.86	2008.89	0.03	15.23
TMB_CYAKI	1903.95	1903.93	0.02	14.29
TMB_CAYKI	1903.95	1904.00	0.05	26.68
TMB_CYRKI	2159.14	2159.19	0.05	23.07
TMB_CYYKL	2180.03	2180.06	0.03	12.02

Supplementary Table 2. Bacterial strains used in this study.

Strain	Description	Reference
W3110	Wild-type	
SK339	W3110 *degP(R207P/Y444A)*	2
SK345	W3110 Δ*degP(orf)*	3
RFM795 (BAS849)[a]	MCR106 *imp*-4213	4
imp4213 Δ*degP*	RFM795 Δ*degP::pheS-kan*^{R}	This study
SK322	X90(DE3) Δ*degP::pheS-kan*^{R}	1

[a] RFM795 is denoted as *imp4213* in this paper

Supplementary Table 3. Plasmids used in this study.

Plasmid	Description	Reference
p7	pET15b-6His_degP(no signal sequence)	1
pSK318	pET22b-degP(full length; S210A)_6His	1
pSK318	pET22b-degP(full length; C57S/C69S/N296C/S210A)_6His	1
pSK387	pET28b-6His_MBP_Tev_lysozymeCS(D18-I58)	1

Supplementary References

1. Kim, S., Grant, Robert A. & Sauer, Robert T. Covalent Linkage of Distinct Substrate Degrons Controls Assembly and Disassembly of DegP Proteolytic Cages. *Cell* **145**, 67-78 (2011).
2. Kim, S. & Sauer, R.T. Distinct regulatory mechanisms balance DegP proteolysis to maintain cellular fitness during heat stress. *Genes Dev.* **28**, 902-911 (2014).
3. Kim, S. & Sauer, R.T. Cage assembly of DegP protease is not required for substrate-dependent regulation of proteolytic activity or high-temperature cell survival. *Proc. Nati. Acad. Sci.* **109**, 7263 (2012).
4. Sampson, B.A., Misra, R. & Benson, S.A. Identification and characterization of a new gene of Escherichia coli K-12 involved in outer membrane permeability. *Genetics* **122**, 491-501 (1989).
