# Supplementary material for: Over-activation of a nonessential bacterial protease DegP as an antibiotic strategy
Source: Commun Biol. 2020 Oct 1;3:547. doi: 10.1038/s42003-020-01266-9 (PMC7529758; doi:10.1038/s42003-020-01266-9)
Supplement: Supplementary file 2 — Description of Additional Supplementary Files [file 42003_2020_1266_MOESM2_ESM.pdf]

Description of Additional Supplementary Files

File Name: Supplementary Data 1
Description: Source data underlying plots shown in figures.
